# 

**Published:** 1912-01

**Authors:** 


					﻿Vol. XXVII
JANUARY, 1912
No. 7
llllf TEXAS . ||
jMEDICA1 ■
“RedBack” JOUk NAL/
PUBLISHED MONTHLY AT AUSTIN. TE	W
F. E. DANIEL, M. D.»’ - - - Editor ai .oprietor.
PRINTED BY THE VON BOECKMANN-; jgWE^ CO|	'
=== JAN. 1Q 1912
Subscription, $1.00 a Year, in Advance. -	-	-	- Single Copies, 10 Cents
I SuRCEtw-CfttSAi's Office
Vmmwi i«i	Mg,, I ..
PnforoH of fl-iA Prwcfrtffinx* at Austin Tovac cprnnd rlfKQ rnA.il matfpr
				

